# Renal impairment in stroke patients: A comparison between the haemorrhagic and ischemic variants

**DOI:** 10.12688/f1000research.12117.2

**Published:** 2017-10-06

**Authors:** Pratyush Shrestha, Shalima Thapa, Shikher Shrestha, Subash Lohani, Suresh BK, Oscar MacCormac, Lekhjung Thapa, Upendra Prasad Devkota

**Affiliations:** 1 National Institute of Neurological and Allied Sciences, Kathmandu, Nepal; 2 Department of Neurosurgery, St Mary’s Hospital, London, W2 1NY, UK

**Keywords:** Estimated Glomerular Filtration Rate, Haemorrhagic, Ischemic, Renal Impairment, Stroke

## Abstract

*Background:* Renal impairment is regularly seen in hospitalized stroke patients, affecting the outcome of patients, as well as causing difficulties in their management. A prospective cohort study was conducted to assess the trend of renal function in hospitalized ischemic and haemorrhagic stroke patients. The incidence of renal impairment in these subgroups, the contributing factors and the need for renal replacement in renal impaired patients was evaluated.

*Methods:* Alternate day renal function testing was performed in hospitalized stroke patients. Estimated glomerular filtration rate (e-GFR) was calculated and the trend of renal function in the two stroke subgroups (haemorrhagic and ischemic) was assessed, with renal impairment defined as e-GFR < 60mL/ minute per 1.73m ^2^.

*Results:* Among 52 patients, 25 had haemorrhagic stroke (mean age 59.81 ± 14.67) and 27 had ischemic stroke (mean age 56.12 ± 13.08). The mean e-GFR (mL/minute per 1.732m ^2^) at admission in the haemorrhagic stroke subgroup was 64.79 ± 25.85 compared to 86.04 ± 26.09 in the ischemic stroke subgroup (p=0.005). Sixteen out of 25 (64%) patients in the haemorrhagic stroke subgroup and 9 out of 27 (33.3%) patients in the ischemic subgroup developed renal impairment (p=0.027). The location of the bleed (p=0.8), volume of hematoma (p=0.966) and surgical intervention (p=0.4) did not predispose the patients to renal impairment. One out of 16 patients with haemorrhagic stroke (who eventually died), and 2 out of 9 patients with ischemic stroke required renal replacement.

*Conclusion*: Renal impairment is commonly seen in stroke patients, more so in patients who suffered haemorrhagic strokes.  The impairment, however, is transient and rarely requires renal replacement therapy.

## Introduction

Renal dysfunction is commonly seen in hospitalized stroke patients. Ischemic stroke is frequently associated with renal dysfunction and nearly a third of patients hospitalized with intracerebral haemorrhage (ICH) have chronic kidney disease (CKD) (estimated glomerular filtration rate [e-GFR] < 60ml/minute per 1.73m ^2^) ^1, 2^. The severity of the impairment and the requirement of renal replacement therapy for these patients in the course of their treatment are important management issues not currently well addressed by the literature. It has been established that good overall medical care can greatly influence the outcome of patients with stroke ^3^. Cerebrovascular accident itself has a high burden of morbidity and mortality, and additionally, CKD is an independent predictor of poor clinical outcome and mortality after an initial stroke ^4, 5^. Co-existence of adverse conditions, such as anemia, oxidative stress, platelet dysfunction, electrolyte imbalance and hyperhomocysteinemia, in patients with CKD have been implicated as the reason why these patients have poorer outcomes compared to the normal population ^6^. Moreover, even mild stages of CKD increases the risk of future ischemic and haemorrhagic strokes ^7^. e-GFR decreased <40mL/min indicates that the risk of symptomatic stroke in the general population is increased by 3.1 times ^8^. Hence proper and routine evaluation of renal function in hospitalized patients with cerebrovascular accident needs to be ensured to improve the outcome of these patients.

Since there is inadequate knowledge concerning the trend of renal function in hospitalized stroke patients and a lack of clear guidelines regarding the need for renal replacement in these type of patients, we conducted an observational study of the trends in renal function in all stroke patients hospitalized at our centre.

## Methods

A prospective study was conducted at the National Institute of Neurological and Allied Sciences, a 100-bed, tertiary care neuro-centre in Kathmandu, Nepal. Stroke patients admitted between December 2014 and April 2015 were recruited to the study. The Institutional Review Board of the National Institute of Neurological and Allied Sciences approved the study (6/2014). The review board confirmed that as this is a descriptive study with no active interventions performed on the patients and their personal identity was not disclosed, no informed consent from the patients was required.

Non-probability, consecutive sampling was performed; all patients with haemorrhagic and ischemic stroke admitted during the study period were enrolled into the study. Patients admitted less than 5 days were excluded as trend analysis was not possible in them. The mean e-GFR at admission and at one week interval was calculated. Serum urea and creatinine were evaluated on alternate days throughout their hospital stay, and the e-GFR was calculated as per the Modification of Diet in Renal Disease Study group formula: e-GFR (mL/minute per 1.732m ^2^) = 186 X [serum creatinine] ^- 1.154^ X age ^- .203^ X [.742 if female] X [1.21 if of African descent] ^9^. Renal impairment was defined when the e-GFR was < 60mL/minute per 1/73m ^2^.

All data were taken from patient hospital records. If data was not available, the information was sought from the patients’ relatives, which was obtained as part of routine care. The primary outcome measure was the occurrence of renal impairment during the hospital stay. The trend of renal function and the need for renal replacement in these patients were analyzed over the duration of admission in the hospital. Demographic, clinical profile and the trend in renal function of patients with haemorrhagic stroke were compared with those of ischemic stroke. The number of patients who had renal impairment according to our criteria was analyzed and any surgical interventions carried out on these patients were recorded. Of the patients that developed renal impairment, the ones that required renal replacement were strictly followed. Since renal replacement is not available at our centre, the patient were monitored when they were moved to nearby hospitals.

Data were entered in IBM SPSS (version 20; SPSS Inc., Chicago, IL, USA) and Pearson's chi square test, Fischer's exact test and independent variable t-test were applied. Line graphs were made to show the trends in the e-GFR in all the patients.

## Results

### Demography

A total of 55 patients were admitted during this period of five months to the intensive care unit and general wards; however, three patients had to be excluded as their hospital stay was less than 3 days. Hence the trend in renal function was followed up in 52 patients – 25 with haemorrhagic stroke and 27 with ischemic stroke. The mean hospital stay of the patients in the haemorrhagic stroke group was 14.58 ± 7.19 days and 9.86 ± 5.12 days in the ischemic stroke group; however, there were two patients from each group who were admitted for more than a month.

The mean age of patients in the haemorrhagic group was 59.81 ± 14.67 and that in the ischemic stroke group was 56.12 ± 13.08, which was comparable. In the haemorrhagic stroke group, 17 were men and 8 were women, while in the ischemic stroke group, 17 were men and 10 were women.

### Role of hypertension

The mean systolic and diastolic blood pressures (mmHg) in the patients with haemorrhagic stroke at admission were 183.6 ± 30.4 and 105.6 ± 18.1, respectively. In the ischemic stroke group, the mean systolic and diastolic blood pressures at admission were 146.9 ± 27.1 and 90.9 ± 14.6, respectively. 23/25 patients with haemorrhagic stroke had hypertension (14 uncontrolled and 9 newly diagnosed); and only two were normotensive. 18/27 patients with ischemic stroke had hypertension (9 controlled, 8 uncontrolled and 1 newly diagnosed) and 9 were normotensive. 10/14 patients with uncontrolled hypertension and haemorrhagic stroke had renal impairment; however, 2/8 patients with uncontrolled hypertension and ischemic stroke had renal impairment. There were 9 patients with ischemic stroke who had controlled hypertension and 4 of them developed renal impairment. No significant association between hypertension and renal impairment was observed (p=0.993; Table 1). The mean arterial pressure at admission in patients who eventually had renal impairment was 124.35 ± 22.60, and in those who did not have renal impairment was 117.32 ± 19.03, which was not statistically significant (p=0.311).

**Table 1.** Association between hypertension and renal impairment in patients who had haemorrhagic or ischemic stroke.

Stroke type			Renal impairment, n		Total, n	Pearson's Chi Square (p-value)
			No	Yes		
Haemorrhagic	hypertension	Absent	1	1	2	0.676
		Uncontrolled	4	10	14	
		Newly diagnosed	4	5	9	
	Total		9	16	25	
Ischemic	hypertension	Absent	6	3	9	0.741
		Controlled	5	4	9	
		Uncontrolled	6	2	8	
		Newly diagnosed	1	0	1	
	Total		18	9	27
Total	hypertension	Absent	7	4	11	0.793
		Controlled	5	4	9	
		Uncontrolled	10	12	22	
		Newly diagnosed	5	5	10	
	Total		27	25	52	

### Analysis of the trend of e-GFR

**Figure 1.**
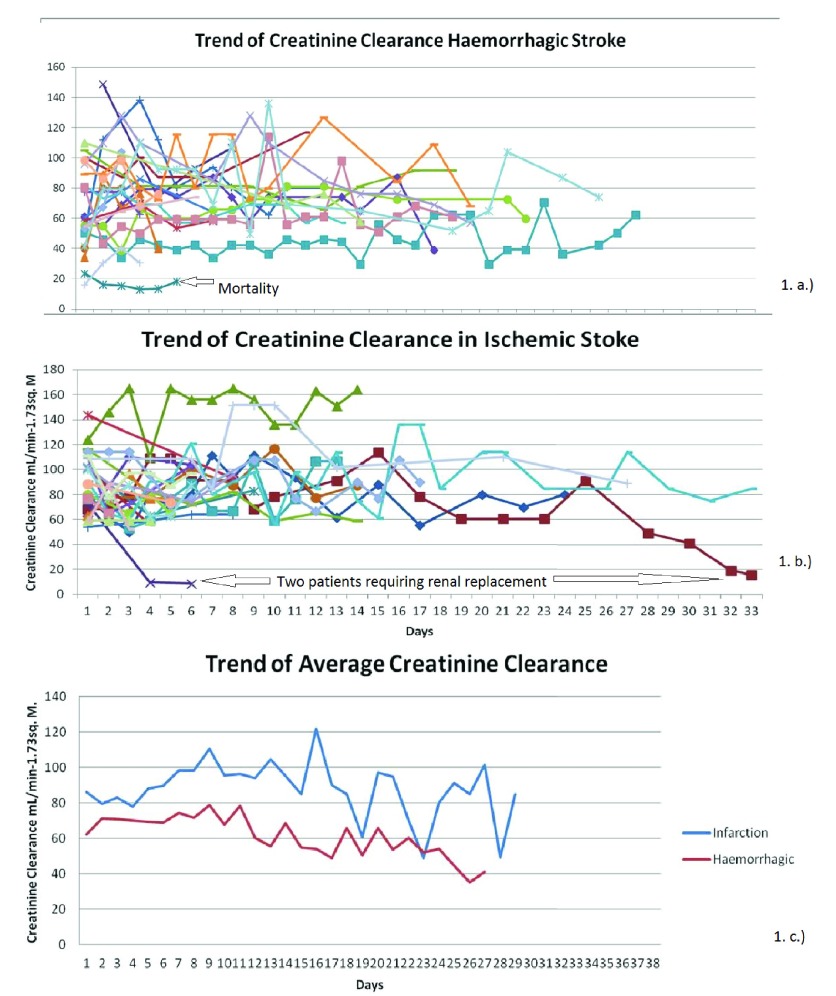
Trends of e-GFR in patients with ( **A**) hemorrhagic and ( **B**) ischemic stroke. ( **C**) Trend of average e-GFR.

The trend in the renal functions in the two groups was plotted over time ( Figure 1). It is evident from this plot that the trend of e-GFR in the ischemic stroke patients is mostly more than 60mL/min per 1.73m ^2^; whereas in the group of patients with haemorrhagic stroke, many of the patients have e-GFR values that demonstrate renal impairment. This was analysed on a two by two table and a Pearson Chi Square test was done to determine if this finding was statistically significant.

Sixteen out of 25 (64%) patients with haemorrhagic stroke and 9 out of 27 (33.3%) patients with ischemic stroke had some grade of renal impairment during their hospital stay, which was statistically significant (Pearson's chi square test, p=0.027; Table 2).

**Table 2.** Crosstablulation of haemorrhagic and ischemic stroke patients with and without renal impairment.

Stroke type	Renal impairment, n			
	No	Yes	Total, n	Pearson chi square test (p-value)
Haemorrhagic	9	16	25	0.027
Ischemic	18	9	27	
Total	27	25	52	

Patients with haemorrhagic stroke presented with an initial lower e-GFR compared to patients with ischemic stroke. The mean e-GFR (mL/minute per 1.732m ^2^) on the day of admission in the haemorrhagic stroke group was 64.79 ± 25.85 compared to 86.04 ± 26.09 in the ischemic stroke group, which was statistically significant (independent sample t-test, p=0.005). However, by the end of a week, the difference was not statistically significant; 80.91 ± 30.74 for the haemorrhagic stroke group and 98.11 ± 31.92 for the ischemic stroke group (independent sample t-test, p=0.294).

### Relationship with volume of blood

The location of the intracerebral bleed in the haemorrhagic stroke group did not predispose the patient to renal impairment (p=0.80; Table 3). In the haemorrhagic stroke group, the mean volume of hematoma in the patients that developed renal impairment was 29.23 ± 24.23 mL and in the patients that did not develop renal impairment it was 28.61± 42.24 mL, which was not statistically significant (p=0.966). Hence, the volume of bleed did not influence the patient developing renal impairment.

**Table 3.** Cross tabulation of haemorrhagic stroke patients with and without renal impairment according to the location of bleed.

Location of bleed	Renal impairment, n		Fisher's test (P-value)
	No	Yes	
Putamen	4	7	0.80
Thalamus	3	6	
Lobar	0	2	
Cerebellar	1	1	
Brainstem	1	0	
Total	9	16	

### Relationship with management

Considering the management of the patients, in the haemorrhagic stroke subgroup, 6 patients underwent evacuation of hematoma, 5 patients underwent CSF drainage procedures and 14 patients were managed conservatively. In the ischemic stroke group, 6 patients underwent decompressive craniectomy, 19 were conservatively managed and 2 patients with cerebellar infarction underwent posterior fossa decompression and evacuation of the infarction.

In the haemorrhagic stroke group, 9 out of 11 patients who underwent surgical intervention developed renal impairment; yet, this finding was not statistically significant (p=0.40; Table 4) Patients requiring surgery were the ones that were more unwell and were likely to be at a higher risk of renal impairment; however, a significant association could not be established ( Table 4).

**Table 4.** Crosstablulation of haemorrhagic and ischemic stroke patients with and without renal impairment according to intervention.

	Intervention	Renal impairment, n		Fisher's test (P-value)
		No	Yes	
Haemorrhagic stroke	Conservative management	7	7	0.40
	Evacuation of hematoma	1	5	
	CSF drainage	1	4	
Ischemic stroke	Conservative management	13	6	
	Decompressive craniectomy	4	2	
	Decompression and evacuation of the infarction	1	1	

### Need of renal replacement

Sixteen out of 25 patients (64%) with haemorrhagic stroke and nine out of 27 patients (33.3%) with ischemic stroke developed some degree of renal impairment. However, only three patients (one with haemorrhagic and two with ischemic stroke) required renal replacement therapy. In the vast majority of the patients, 22 out of 25 (88%), the renal impairment was transient and improved without hemodialysis.

The patient who underwent renal replacement therapy after haemorrhagic stroke, was a 45 year old, who had 75mLs of ganglionic bleed evacuated, and required hemodialysis from the 5 ^th^ day of admission. This patient succumbed to multiple organ dysfunction syndrome on the 14 ^th^ day. The two patients with ischemic stroke who required renal replacement on the 4 ^th^ and 31 ^st^ day of admission improved with hemodialysis.

## Discussion

Renal failure is a rare primary cause of death in patients following stroke; however, impaired renal function is a significant predictor of both short and long term mortality in these patients. The Oxford Community Stroke Project reported that stroke survivors tend to die 2.3 times more than the matched general population every year post-stroke, and non-stroke cardiovascular diseases is the most common cause of death following the first year of initial stroke ^10^. Calculated e-GFR ≥ 51.27ml/min predicts a better long term survival in stroke patients after adjustment of confounders (age, neurological state, ischemic heart disease, hypertension, smoking and diuretic use). MacWalter *et al.* suggested the use of ACE inhibitors in this subgroup of patients, as they had higher risk of mortality ^11^.

Hypertension is attributed to a large share of patients progressing to end stage renal failure ^12^. In the present study, 23 out of 25 patients with haemorrhagic stroke had hypertension (14 uncontrolled and 9 newly diagnosed). However, 18 out of 27 patients with ischemic stroke had a normal blood pressure (9 without history of hypertension and 9 controlled). Even the patients with well-regulated blood pressure continued to have renal impairment, i.e. 5 out of 9 patients. Additionally, 4 out of 11 patients without hypertension, 4 out of 9 patients with controlled hypertension, 12 out of 22 patients with uncontrolled hypertension and 5 out of 10 patients with newly diagnosed hypertension developed renal impairment, which was not statistically significant (independent t-test, p=0.793). Therefore, in our patients, hypertension did not play a significant role in predisposing patients to renal impairment.

In our study, some degree of renal impairment was seen in 64% of patients with haemorrhagic stroke and 33.3% of patients with ischemic stroke. Ovbiagele *et al.* have reported renal impairment in nearly a third of patients with ICH; and Mishra and colleagues in 38.46% of such patients ^1, 13^. Use of mannitol could be a confounding variable in our study. Dziedzic *et al*. found that the use of mannitol decreased intra cranial pressure, caused transient elevation of urea and creatinine, but did not cause anuria or oliguria ^14^. The higher incidence of renal impairment seen in the haemorrhagic stroke subgroup may be due to the inhererent difference in the management of haemorrhagic and ischemic stroke, in regards to the use of mannitol, nephrotoxic drugs, various antibiotics, etc. This study is unable to explore this possibility in detail; however, as all these patients were managed in the same institution, over the same period of time, and the baseline management in both these groups is grossly similar, we found it interesting to highlight this difference. As ours is a tertiary care centre receiving referred cases from all over the country, selected severe patients could have been enrolled into our study. Of 614,454 patients admitted with ICH in hospitals in the USA between 2005 and 2011, 41694 (6.8%) had acute renal failure (ARF), with 700 patients (1.7%) requiring in-hospital dialysis. These patients with ARF had higher rates of moderate-to-severe disability and in-hospital mortality ^15^.

After excluding patients with a history of renal disease, dehydration, nephrotoxic drugs use or septicemia, transient renal impairment was found in 30 of 78 patients (38.46%) with acute ICH. Renal functions normalized in 2–4 weeks ^15^. Hence, only a small percentage of patients with ICH who develop renal impairment, need renal replacement. Only one of the 25 patients with ICH and two of the 27 patients with ischemic stroke required renal replacement in our study. This finding is coherent with Saheed *et al.*, who found that of only 700 (1.7%) of 41, 694 patients with some renal impairment requiring renal replacement in 614,454 patients with ICH admitted in the USA ^15^. The Oxford Community Stroke Project further states that renal failure is a rare primary cause of death in post-stroke patients, but is a significant predictor of increased mortality in both the short and long term ^10, 16^.

The size of the hematoma did not predispose patients to renal impairment in our study, as the average hematoma volume in the group of patients that developed renal impairment was 29.23 ± 24.23mL and in the patients that did not develop renal impairment was 28.61± 42.24 (p=0.966). In a similar study by Cutting *et al.* (2004), there was no correlation between admission GFR and ICH volume (p=0.77), and patients with GFR <60 versus patients with GFR ≥ 60mL/min/1.73 m ^2^ also had similar ICH volumes (median 10.8mL vs 11.4mL; p=0.54) ^17^. However, Molshatzki and colleagues (2011) were of the opinion that patients with larger ICH were predisposed to having more severe renal impairment. In their no renal impairment group, mild impairment group and the moderate to severe impairment group, the median hematoma volumes were 15.3mL, 16.6mL and 50.2mL, respectively (p=0.009) ^18^. In addition, patients with moderate/severe impairment exhibited a 2.3-fold higher hematoma volume than the other groups (p=0.04). In our study, the location of the hematoma did not have any association with the severity of the renal impairment. Molshatzki *et al.* concluded that patients with moderate to severe renal impairment had a > 6-fold higher odds of lobar location of the bleed (95% CI = 1.59-24.02) as compared to the patients with no impairment.

The mean e-GFR (mL/minute per 1.732m ^2^) on the day of admission in the haemorrhagic stroke group was significantly lower (64.79 ± 25.85) compared to the ischemic stroke group (86.04 ± 26.09) in our study. In Hao *et al.’s* (2010) study, the mean e-GFR at admission in the haemorrhagic and ischemic stroke groups were comparable 77.57 ± 51.73 vs 77.07 ± 29.89 (p=0.285) ^19^. Patients presented to our centre at various times following the index stoke; hence, the days mentioned are not the days after the stroke event, but the days after admission to the hospital. This is a limitation of our study as the values mentioned do not reflect the trend profile of renal parameters following stroke.

Small vessel disease is a generalized condition affecting the vascular beds in the brain, kidneys and retina. Makin and colleagues (2015) tried to study the relation of small vessel (lacunar) stroke with renal impairment with the hypothesis that lacunar infarction would be associated with more renal impairment compared to cortical stroke; however, there was no such difference ^20^. We are not able to comment on this as we have not categorized ischemic stroke into these subgroups in this study.

## Conclusions

Renal impairment is commonly seen in stroke patients and is more common in the haemorrhagic variety, particularly in the initial days of the ictus. The impairment however is transient and rarely requires renal replacement therapy. The volume of the intracerebral bleed, its location and whether the patients were surgically or conservatively managed did not predispose them to renal impairment.

## Data availability

The data referenced by this article are under copyright with the following copyright statement: Copyright: © 2017 Shrestha P et al.

Raw data for renal impairment in stroke patients is available from http://dx.doi.org/10.7910/DVN/EILQSZ ^21^.

Coding schema for Excel spreadsheet (schema included in SPSS version): SN, Serial Number; age (1, <20; 2, 21–30; 3, 31–40; 4, 41–50; 5, 51–60; 6, 61–70; 7, 71–80; 8, 81–90); sex (0- female, 1-male); location, location of the bleed; IVE, intraventricular extension (1-yes, /- no); Renal_impair, patients that developed renal impairment (1-yes, 0-no); Renal_replace, patients that required renal replacement (1-yes, 0-no); Volume, volume of bleed in millilitres; htnhth, hypertension (0-absent, 1-controlled, 2-uncontrolled, 3-newly diagnosed), sbp, systolic blood pressure in mmHg at admission; dbp, diastolic blood pressure in mmHg at admission; MAP, mean arterial pressure at admission; operation (EVD, external ventricular drain; VP shunt, ventriculoperitoneal shunt; evacuation, evacuation of bleed or infarction); interv, intervention (1, conservative management (haemorrhagic); 2, evacuation of hematoma; 3, CSF drainage; 4, conservative (Infarction); 5, decompressive craniectomy; 6, decompression and evacuation of the infarction); egfr1, estimated glomerular filtration rate at day 1; Meaneffr, mean egfr of each patient; Meandrop, maximum drop in egfr during the hospital stay of the patient as compared to the day of admission.

NOTE: The hospital stay can be determined by counting the number of egfrs recorded.
