# Supplementary material for: Renal impairment in stroke patients: A comparison between the haemorrhagic and ischemic variants
Source: F1000Res. 2017 Oct 6;6:1531. Originally published 2017 Aug 21. [Version 2] doi: 10.12688/f1000research.12117.2 (PMC5615769; doi:10.12688/f1000research.12117.2)
Supplement: Peer review history

# Peer review: Referee response for version 2

**DOI:** 10.5256/f1000research.13947.r26792

**Published:** 2017-10-18

**Authors:** Ravi Dadlani

**Affiliations:** 1 Department of Neurosurgery, Apollo Specialty Hospital, Nellore, Andhra Pradesh, India

The authors have made adequate changes in the discussion in the article. It may be accepted for indexing.

I have read this submission. I believe that I have an appropriate level of expertise to confirm that it is of an acceptable scientific standard.

# Peer review: Referee response for version 1

**DOI:** 10.5256/f1000research.13115.r25450

**Published:** 2017-09-25

**Authors:** Dibya Singh Shah

**Affiliations:** 1 Department of Nephrology, Tribhuvan University Teaching Hospital, Kathmandu, Nepal

As we have very few literature and data in Nepalese populations in this field, this study is a good initiation. In this study eGFR at presentation was lower in hemorrhagic stroke group as compared to ischemic stroke. One explanation could be due to much higher blood pressure at presentation in this population as  compared to ischemic stroke. As this is a small single center study larger multicentre studies are needed to draw further conclusion.

I have read this submission. I believe that I have an appropriate level of expertise to confirm that it is of an acceptable scientific standard.

# Peer review: Referee response for version 1

**DOI:** 10.5256/f1000research.13115.r25749

**Published:** 2017-09-22

**Authors:** Umit Eroglu, Fatih Yakar

**Affiliations:** 1 Department of Neurosurgery, Faculty of Medicine, Ankara University, Ankara, Turkey; 2 Kars harakani Hospital, Kars, Turkey

The authors searched renal impairment rate among stroke patients. That’s a valuable study especially for ICU units of neurology and neurosurgery departments.

 Authors evaluated hematoma volume, surgical interventions and hematoma location. But ventricular hematoma can cause biochemical changes so this condition had to be evaluated separately.

 Cholesterol level, alcohol intake, smoking, seizures (status) and antiepileptic drugs could be the other parameters for haemorrhagic stroke.

 It is appropriate to index the article in this state.

We have read this submission. We believe that we have an appropriate level of expertise to confirm that it is of an acceptable scientific standard.

# Peer review: Referee response for version 1

**DOI:** 10.5256/f1000research.13115.r25747

**Published:** 2017-09-20

**Authors:** Ravi Dadlani

**Affiliations:** 1 Department of Neurosurgery, Apollo Specialty Hospital, Nellore, Andhra Pradesh, India

This is a very interesting study. 

 I congratulate the authors on this unique analysis. 

 I, however, have found some issues with the data. 

 The hemorrhagic group appears to have more statistically significant 

 renal impairment than the ischemic group. 

 Rather than comparing data with regards to blood clot volume, surgery etc.,

 It would be more pertinent to look at the basic management for these patients

 in terms of intravenous fluids, mannitol, glycerol, types of maintenance IV fluids, insensible 

 los (fever etc.,), Nephrotoxic drugs, antibiotics used etc.,

 If the above mentioned analysis is added to the paper, it may have some value. 

 Also, then, specific recommendations may be given regarding the overall management of such patients. 

 I believe the differences in the two groups is artificial because the general management differs. 

 I would not recommend the paper be published in it's present form.

I have read this submission. I believe that I have an appropriate level of expertise to state that I do not consider it to be of an acceptable scientific standard, for reasons outlined above.

# Response

**Published:** 2017-10-03

**Authors:** Pratyush Shrestha

**Affiliations:** National Institute of Neurological and Allied Sciences, Nepal

We would like to thank our referee for finding our study interesting, the analysis unique and most important of all, giving us his precious time.

We agree to his suggestion that it would be more pertinent to look at the basic management for these patients in terms of IV fluids, mannitol, insensible loss, use of antibiotics and nephrotoxic drugs. We could probably continue to work on these issues in our future publications.

We, however, disagree to his claim that the difference in this two groups is artificial and is due to the difference in the general management.

We have calculated the mean e-GFR at admission for both the groups. The e-GFR in the haemorrhagic stroke group is significantly low compared to the ischemic stroke subgroup.

***"The mean e-GFR (mL/minute per 1.732m ^2^) at admission in the haemorrhagic stroke subgroup was 64.79 ± 25.85 compared to 86.04 ± 26.09 in the ischemic stroke subgroup (p=0.005)*"**

We agree that the management of haemorrhagic stroke and ischemic stroke differs. 

But we would like to stress that these subgroups were patients admitted in the same unit, over the same period of time;and, on the whole the baseline management in both the groups, in regards to the IV fluids used, the use of mannitol, insensible loss, use of antibiotics and nephrotoxic drugs  was grossly indifferent.

We have included this issue in our discussion and Dziedzic *et. al*. did find that mannitol caused transient elevation of urea and creatinine, but did not cause anuria or oliguria.

Finally, we would like to thank our referee once again for his valuable suggestions.
